# Supplementary material for: Latest advancements and prospects in the next-generation of Internet of Things technologies
Source: PeerJ Comput Sci. 2024 Oct 25;10:e2434. doi: 10.7717/peerj-cs.2434 (PMC11622910; doi:10.7717/peerj-cs.2434)
Supplement: Table S1 [file peerj-cs-10-2434-s001.docx]

| **Challenges** | **Contributions** | **Future research directions and remedies** |
| --- | --- | --- |
| Complexity | [69] | - How to solve the complexity issue in IoT? - The complex network-based approaches are soultions. |
| Connectivity | [71] | - How to connect a large number of objects in IoT-based technologies? - The usage of social relations and exploring AI and machine learning. |
| IoT and SIoT standards | [72] | - There is a lack of IoT and SIoT standards. - The development of new standards combining complex networks is a good research direction. |
| Complex data | [73] | - How to handle diverse data types in IoT? - The use of AI and Machine learning is helpful to overcome this issue. |
| Noisy data | [75] | - How to clean noisy big data? - Develop more advanced AI-based methods. |
| Self-organized structures | [78] | - How to handle self-organization and autonomous IoT infrastructure? - Develop novel methods using learning systems and automated reasoning. |
| Trust, security, and privacy | [80] | - What entities are available in the IoT-based technologies? - How is the trust management process more efficient and accurate? - Are any efficient trust models available in the IoT? - What models and tools are available for security and privacy? |
| Energy Management | [83] | - What are possible solutions for efficient energy management in IoT-based technologies? |
